# Diabetic Macular Edema

**DOI:** 10.12669/pjms.322.8496

**Published:** 2016

**Authors:** Fatih C. Gundogan, Umit Yolcu, Fahrettin Akay, Abdullah Ilhan, Gokhan Ozge, Salih Uzun

**Affiliations:** 1 Fatih C. Gundogan, GATA Medical School, Ophthalmology, Ankara, Turkey; 2 Umit Yolcu, Sarikamis Military Hospital, Ophthalmology, Kars, Turkey; 3 Fahrettin Akay, İzmir Military Hospital, Ophthalmology, Izmir, Turkey; 4 Abdullah Ilhan, Erzurum Military Hospital, Ophthalmology, Erzurum, Turkey; 5 Gokhan Ozge, GATA Medical School, Ophthalmology, Ankara, Turkey; 6 Salih Uzun Etimesgut Military Hospital, Ophthalmology, Ankara, Turkey

**Keywords:** Diabetic macular edema, Optical coherence tomography, Fluorescein angiography, Ranibizumab, Bevacizumab, Triamcinolone acetonide, Pars plana vitrectomy

## Abstract

Diabetic macular edema (DME), one the most prevalent causes of visual loss in industrialized countries, may be diagnosed at any stage of diabetic retinopathy. The diagnosis, treatment, and follow up of DME have become straightforward with recent developments in fundus imaging, such as optical coherence tomography. Laser photocoagulation, intravitreal injections, and pars plana vitrectomy surgery are the current treatment modalities; however, the positive effects of currently available intravitreally injected agents are temporary. At this point, further treatment choices are needed for a permanent effect.

**Sources of data selection:**

The articles published between 1985-2015 years on major databases were searched and most appropriate 40 papers were used to write this review article.

## INTRODUCTION

Diabetic macular edema (DME), one of the major complications of diabetic retinopathy (DRP), is also one of the leading causes of visual impairment in the working-age population.^1^ DME occurs in nearly 12% of patients with DRP and causes more than 10,000 new cases of blindness per year.^2^ Duration and type of diabetes directly affect the prevalence rate of DME. Patients can develop DME in the first five years following diagnosis of type I diabetes. The prevalence rate gradually reaches up to 40% within 30 years.^3^, ^4^ About 5% of patients with type II diabetes already have DME at the time of diagnosis. Duration of diabetes, proteinuria, gender, cardiovascular disease, high levels of HbA1c, and use of diuretics are defined as systemic risk factors.^4^ DME can occur at any stage of DRP.

## PATHOGENESIS

The pathogenesis of DME has not been thoroughly defined because there are complex processes with various contributing factors. Chronic hyperglycemia, hypercholesterolemia, free oxygen radicals, advanced glycation end-products, and protein kinase C are involved in the pathologic process.^5^ The common characteristic is the increase in levels of vascular endothelial growth factor (VEGF), which is responsible for the disruption of the inner blood–retinal barrier (BRB).^6^ Disruption of the BRB leads to the accumulation of subretinal and intraretinal fluid, which in turn alters the macular structure and function. Leukocyte chemotaxis to vascular endothelium is another element of BRB breakdown and vascular leakage.^7^ Leukocytes induce endothelial dysfunction and capillary non-perfusion in multiple ways, such as disruption of tight junctions and free oxygen radicals. DRP stimulates the expression of adhesion molecules that facilitate the adhesion capacity of inflammatory cells; this stimulation is thought to be mediated by VEGF.^8^ It also has been reported that VEGF is responsible for neuronal apoptosis.^9^ Elevated VEGF levels can cause vascular leakage and BRB breakdown, directly and indirectly, via various mechanisms. Nitric oxide is another critical mediator in the pathogenesis of DME. It uses the similar metabolic methods, such as the induction and retention of leukocytes, increased secretion of VEGF, and dysfunction in cellular junctions.^3^ As the primary factor of DRP, hyperglycemia causes tissue edema, perfusion insufficiency, vascular dilation-elongation, BRB breakdown, and neuronal apoptosis, and it certainly takes part in the pathogenesis of DME. The vitreous humor may also be a source of proinflammatory cells and cytokines.^10^ In addition, the presence of vitreoretinal interface pathologies, such as epimacular membranes, can apply tractional forces to the macula and aggravate DME. Hypoxia, ischemia, genetic factors, and inflammatory mediators may contribute to the pathogenesis of BRB damage and DME. In the course of DME, Muller glial cell and pericyte dysfunction can be involved.

The β-adrenergic system is known for its particular roles in angiogenesis and neuronal damage, even in ocular tissues.^11^ Hypoxia may lead to catecholaminergic discharge, and catecholamines induce VEGF and hypoxia-inducible factor-1α (HIF-1α) expression via β-adrenergic receptors, while β-blockers reduce their expression and vascular leakage.^12^ β-1 and β-3 adrenergic receptors are present in retinal endothelial cells. Stimulation of β-3 adrenergic receptors triggers proliferation and migration of retinal and choroidal endothelial cells.^13^ In short, the β-adrenergic system induces both the secretion of proangiogenic factors and endothelial cell responses to form new vessels. These data suggest that the β-adrenergic system might be involved in the DRP process.^14^

Various studies have recently been performed to understand the emerging role of microRNAs in diabetes and cardiovascular diseases.^15^, ^16^ MicroRNAs are short, single-stranded, non-coding RNA sequences that bind to complementary sequences of target mRNAs and act as post-transcriptional regulators. Their activity usually induces gene silencing and prevents protein production. They are involved in multiple physiological and pathological mechanisms, including angiogenesis, diabetes, and cardiovascular diseases. For instance, they take part in pancreatic β-cell differentiation, insulin secretion, and insulin sensitivity.^17^ It has been reported that microRNAs are involved in retinal and choroidal neovascularization via down regulation of VEGF.^18^ Although not enough studies have been conducted regarding the role of microRNAs in DRP to come to distinct conclusions, there is strong evidence of their contribution.^17^ Different microRNAs have been defined that affect endothelial cell proliferation, apoptosis, VEGF expression, or neuronal damage during the course of DME. MicroRNA agonists or antagonists may constitute alternative treatment options in the management of DRP and its major complication, DME.

## DIAGNOSIS AND CLASSIFICATION

**Fig.1:**
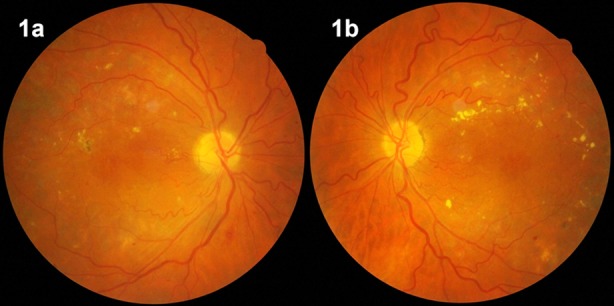
Diabetic macular edema in the right (1a) and left eye (1b) of a patient.

**Fig.2:**
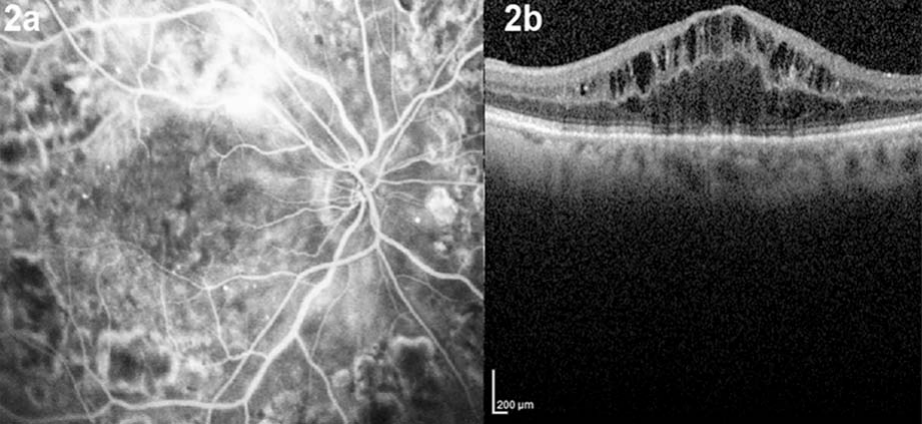
Fundus fluorescein angiography (FFA) (2a) and optical coherence tomography (2b) images of diabetic macular edema. Fluorescein leakage (2a) and cystoid spaces (2b) are seen in FFA and OCT, respectively.

The first step in the diagnosis of DME is the contact lens-aided slit-lamp biomicroscopy of the posterior pole. Biomicroscopic examination reveals the presence and location of macular thickening, exudates, and cystoid changes (Fig.1). Fundus fluorescein angiography (FFA) may show the areas of retinal capillary leakage (Fig.2a), and optical coherence tomography (OCT) can show retinal section images. With the aid of OCT, qualitative and quantitative evaluations of structural changes in the macula have become prominent in the diagnosis (Fig.2b), treatment, and follow up of DME.

**Fig.3:**
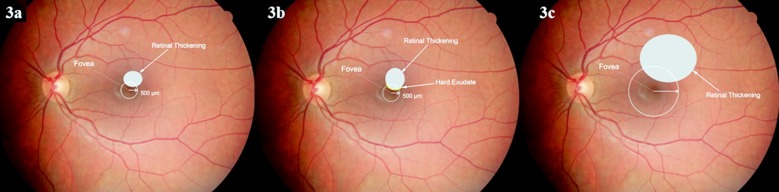
Schematic view of clinically significant macular edema.

DME can be associated with or without exudates and can appear as focal, diffuse, and ischemic subtypes. Focal macular edema is characterized by fluorescein leakage from certain capillary areas, and it may be surrounded by hard exudates (circinate exudates) that are yellow to white in color and contain lipoprotein remnants of plasma extravasation from microaneurysms. The diffuse form is the result of generalized capillary dysfunction and BRB disruption. Cystoid macular edema usually comes along with diffuse DME.^19^ Ischemic DME, which results from microvascular occlusions in any part of the macular area, is observed in FFA as hypofluorescent macular areas. Fig.3 shows a fundoscopic view and FFA of a diabetic retinopathy patient with focal macular edema.

**Fig.4:**
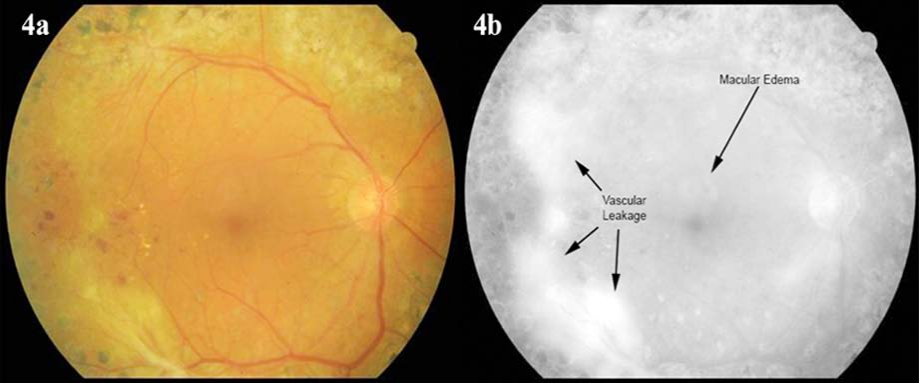
Fundoscopic view (4a) and fluorescein angiography (4b) of a diabetic retinopathy patient with vascular leakage in the temporal retina and focal macular edema.

In the past, the term “clinically significant macular edema” (CSME) was used to define patients who needed to be treated. CSME was identified in the presence of any of the following three fundoscopic examination findings. (Fig.4)^20^

1. Retinal thickening at or within 500 µ or 1/3 the disc diameter of the fovea.
2. Hard exudates at or within 500 µ of the fovea, with adjacent retinal thickening.
3. Retinal thickening greater than one disc diameter (1500 µ) in size that is within one disc diameter from the fovea.

However, these criteria were mostly discarded due to the emergence of OCT-based diagnosis. OCT images are now used in the diagnosis, treatment, and follow up of DME.^3^

## MANAGEMENT STRATEGIES

To prevent the development and progression of DME, it is essential to maintain strict glycemic control, reduce blood lipid levels, and regulate systemic blood pressure. The American Diabetes Association recommends that HbA1c levels must be kept under 7%, blood pressure under 130/80 mmHg, and total lipids under 100 mg/dL.^21^ Ocular treatments include retinal laser photocoagulation, intravitreal administration of certain agents, and vitreoretinal surgery when necessary.

### Laser Treatment

Laser treatment strategies have been derived mainly from the Early Treatment Diabetic Retinopathy Study (ETDRS),^20^ a randomized, prospective clinical trial that evaluated photocoagulation in patients with diabetes in the USA.^20^, ^22^ The ETDRS revealed that cases of CSME treated by laser photocoagulation showed improved visual acuity, reduced risk of visual loss, and negligible visual field loss. Eyes without CSME did not benefit from laser photocoagulation. Some recently published studies still emphasize the superiority of laser photocoagulation compared to other options.^23^ Therefore, it is accepted as the current standard treatment for DME by many authors.^24^-^26^

Some complications associated with laser photocoagulation have been reported, such as macular hemorrhage, choroidal neovascularization, decline in visual acuity and contrast sensitivity, and visual field defects.^27^ The aim of laser photocoagulation is to destroy the outer segments of photoreceptors and diminish oxygen consumption; in order to minimize this effect, several attempts have been made to optimize the parameters and application techniques.^28^

### Anti–VEGF Agents

Since the approval of bevacizumab for colorectal cancer in 2004 and ranibizumab for age-related macular degeneration in 2006, the number of ophthalmology fields in which anti-VEGF agents are used has been increasing. Pegaptanib, bevacizumab, ranibizumab, aflibercept, and KH902 are the agents currently used for retinal pathologies.^29^-^31^ Each agent has been the subject of several studies and has improved DME considerably. Currently, ranibizumab is the only agent approved by the US Food and Drug Administration for DME. Aflibercept, known as a “VEGF trap” because of its ability to deactivate all six VEGF proteins, is approved for use in wet age-related macular degeneration, but not yet in DME. Recent studies have reported better visual and anatomical results using aflibercept than using standard laser treatments, so it seems to be a promising agent.^32^

**Fig.5:**
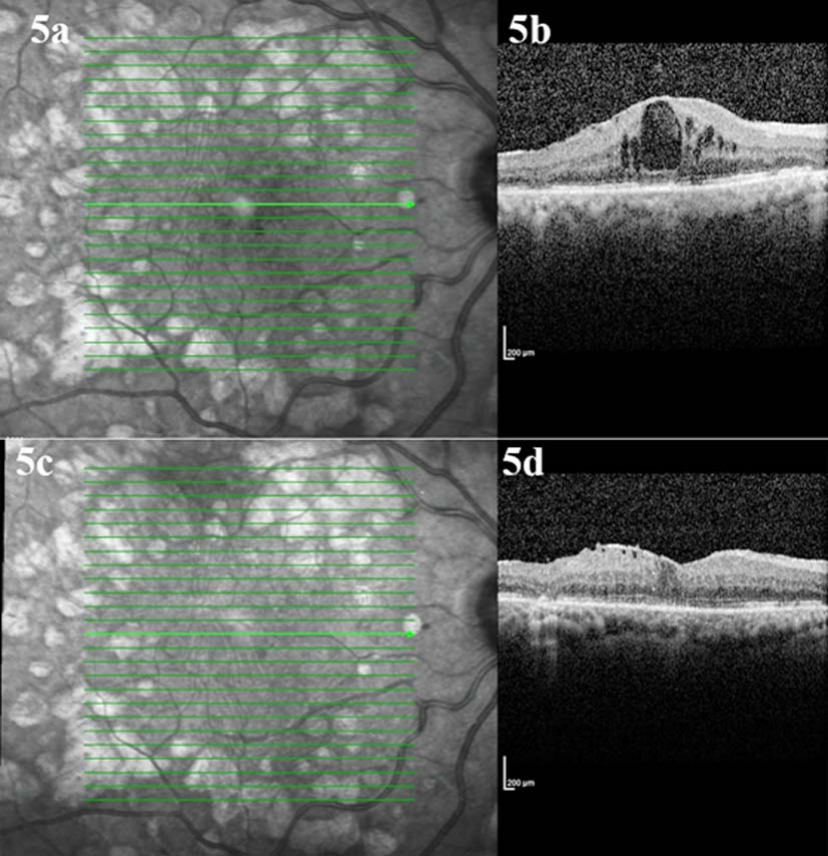
Optical coherence tomography images before the ranibizumab injection (5a, 5b) and after three monthly injections of ranibizumab (5c, 5d).

Ranibizumab, a humanized monoclonal antibody fragment obtained from a mouse monoclonal anti-VEGF antibody, can interfere with all metabolic activities of VEGF. It has also proved to be effective in reducing macular thickness (Fig.5).^33^ A 12-month, randomized, double-masked, multicenter, laser-controlled phase III study (the RESTORE study) was carried out to demonstrate the effects of ranibizumab, laser, and a combination of the two. The study showed that ranibizumab monotherapy and a combination of ranibizumab with laser therapy were more advantageous than laser treatment alone in terms of macular thickness and visual acuity gain. In addition, ranibizumab was well tolerated and did not pose any safety concerns during a three-year follow up.^34^

Anti-VEGF agents are also used in relapsed DME cases. Gulkilik et al. reported the effectiveness of bevacizumab in cases with recurrent DME after pan retinal photocoagulation.^35^ Yuksel et al. reported that intravitreal bevacizumab treatment is effective in cases unresponsive to focal laser photocoagulation or/and subtenon or intravitreal steroid injection.^36^ In some patients, response to intravitreal ranibizumab or bevacizumab may be incomplete or absent.^37^ It has been shown that switching to aflibercept provides anatomical and visual improvement in these cases due to the differences in pharmacodynamics of the drugs.^37^

### Intravitreal Corticosteroids

The benefits of glucocorticoids depend on their anti-inflammatory and anti-VEGF effects.^6^ Triamcinolone acetonide (TA) has been used for various ocular inflammatory disorders, including DME; several studies have considerable improvements in DME with TA alone or combined with anti-VEGF agents.^38^-^41^ Zhang et al. and Qi et al. reported significant visual improvements and decreased central macular thickness, particularly in the early post-injection period. However, the sustainable effects of TA are controversial, especially in terms of anatomical results.^41^, ^42^ Elevated intraocular pressure (IOP) and cataract formation are the main complications of TA injection. While increased IOP sometimes requires medical treatment, only 2% of treated eyes in a previous study needed surgical intervention to reduce IOP.^43^ Fluocinolone acetonide and dexamethasone implants are new options found to be efficient in various studies.^44^, ^45^

### Pars Plana Vitrectomy

Clinical trials have shown that performing pars plana vitrectomy (PPV) in selected DME cases reduced macular edema.^46^ PPV removes traction forces and proinflammatory substances and increases the oxygenation of inner retinal layers.^47^ The procedure can be performed when DME is resistant to laser treatment and anti-VEGF injections. It reduces macular thickness and provides visual acuity gain, and the effects are mostly sustainable.^48^ Better surgical outcomes have been achieved by combining medical and surgical interventions.^49^ The presence of hard exudates, vitreous hemorrhage, and vitreomacular traction may be considered as indications for PPV in DME cases.^46^

## CONCLUSION

DME is a serious public health problem that remains the leading cause of visual impairment in the industrialized world, despite innovations in its diagnosis and treatment. Medical difficulties and excessive economic burdens have forced scientists to seek new options for its treatment. While the discovery of the roles of VEGF and BRB breakdown in DME pathogenesis has accelerated therapeutic research, there are still many challenging cases that are refractory to all treatment efforts.
